# Hospitalisation without delirium is not associated with cognitive decline in a population-based sample of older people—results from a nested, longitudinal cohort study

**DOI:** 10.1093/ageing/afab068

**Published:** 2021-05-03

**Authors:** Sarah J Richardson, Rachael Lawson, Daniel H J Davis, Blossom C M Stephan, Louise Robinson, Fiona E Matthews, Carol Brayne, Linda E Barnes, John-Paul Taylor, Stuart G Parker, Louise M Allan

**Affiliations:** Translational and Clinical Research Institute, Biomedical Research Building, Campus for Ageing & Vitality, Newcastle University, Newcastle upon Tyne NE4 5PL, UK; NIHR Newcastle Biomedical Research Centre, Faculty of Medical Sciences, Newcastle University and Newcastle upon Tyne Hospitals NHS Foundation Trust; Translational and Clinical Research Institute, Biomedical Research Building, Campus for Ageing & Vitality, Newcastle University, Newcastle upon Tyne NE4 5PL, UK; MRC Unit for Lifelong Health and Ageing at UCL, London WC1E 7HB, UK; Institute of Mental Health, Division of Psychiatry and Applied Psychology, School of Medicine, Nottingham University, Nottingham NG7 2TU, UK; Population Health Sciences Institute, Campus for Ageing & Vitality, Newcastle University, Newcastle upon Tyne NE4 5PL, UK; Population Health Sciences Institute, Campus for Ageing & Vitality, Newcastle University, Newcastle upon Tyne NE4 5PL, UK; Cambridge Public Health, University of Cambridge, Cambridge CB2 0SR, UK; Cambridge Public Health, University of Cambridge, Cambridge CB2 0SR, UK; Translational and Clinical Research Institute, Biomedical Research Building, Campus for Ageing & Vitality, Newcastle University, Newcastle upon Tyne NE4 5PL, UK; NIHR Newcastle Biomedical Research Centre, Faculty of Medical Sciences, Newcastle University and Newcastle upon Tyne Hospitals NHS Foundation Trust; Population Health Sciences Institute, Campus for Ageing & Vitality, Newcastle University, Newcastle upon Tyne NE4 5PL, UK; Centre for Research in Ageing and Cognitive Health, University of Exeter, Exeter EX1 2LU, UK

**Keywords:** delirium, cognitive outcomes, cohort study, hospitalised older adults

## Abstract

**Background:**

Acute hospitalisation and delirium have individually been shown to adversely affect trajectories of cognitive decline but have not previously been considered together. This work aimed to explore the impact on cognition of hospital admission with and without delirium, compared to a control group with no hospital admissions.

**Methods:**

The Delirium and Cognitive Impact in Dementia (DECIDE) study was nested within the Cognitive Function and Ageing Study II (CFAS II)–Newcastle cohort. CFAS II participants completed two baseline interviews, including the Mini-Mental State Examination (MMSE). During 2016, surviving participants from CFAS II–Newcastle were recruited to DECIDE on admission to hospital. Participants were reviewed daily to determine delirium status.

During 2017, all DECIDE participants and age, sex and years of education matched controls without hospital admissions during 2016 were invited to repeat the CFAS II interview. Delirium was excluded in the control group using the Informant Assessment of Geriatric Delirium Scale (i-AGeD). Linear mixed effects modelling determined predictors of cognitive decline.

**Results:**

During 2016, 82 of 205 (40%) DECIDE participants had at least one episode of delirium. At 1 year, 135 of 205 hospitalised participants completed an interview along with 100 controls. No controls experienced delirium (i-AGeD>4). Delirium was associated with a faster rate of cognitive decline compared to those without delirium (β = −2.2, *P* < 0.001), but number of hospital admissions was not (*P* = 0.447).

**Conclusions:**

These results suggest that delirium during hospitalisation rather than hospitalisation per se is a risk factor for future cognitive decline, emphasising the need for dementia prevention studies that focus on delirium intervention.

## Key Points

- Hospital admission without delirium was not associated with cognitive decline compared with a control group without hospitalisation.
- Delirium during hospital admission was associated with significant cognitive decline.
- These results suggest that delirium may be a key component of the cognitive decline observed after hospitalisation.
- These results emphasise the need for dementia prevention studies that focus on delirium intervention.

## Introduction

There is much interest in the impact of hospitalisation on older people, particularly in respect of cognition. In a number of large studies, acute hospitalisation has been shown to be adversely associated with increased risks of incident dementia and accelerated trajectories of cognitive decline [1–6]. The components driving these associations are unclear but factors demonstrated to confer increased risk of worse cognitive outcomes following hospitalisation include non-elective admission [1, 2], critical illness [3, 4], admissions for medical conditions [2] and stroke [5], longer admissions [4] along with a number of baseline comorbidities [6].

Delirium is emerging as a significant independent risk factor for future cognitive impairment [7, 8], but the contribution of delirium to the cognitive decline described following hospitalisation has not previously been considered [6]. It is possible that much of the relationship between hospitalisation and cognitive decline is attributable to delirium. Advancing our understanding of the components driving the adverse impact on cognition of hospitalisation, and the potential contribution of delirium, will facilitate the design of targeted interventions to prevent dementia. Delirium is an attractive potential target given that it has been shown to be potentially modifiable [9].

We set out to quantify the relative contribution of hospitalisation per se and delirium episodes on cognitive outcomes in a prospective, longitudinal cohort. In secondary analysis using data from the Delirium and Cognitive Impact in Dementia (DECIDE) study, a population-based study of cognitive outcomes in older people following delirium, we compared cognitive outcomes in those hospitalised with and without delirium, with a control group with no hospital admissions. We hypothesised that people who were hospitalised and did not have delirium would not differ in their cognitive trajectory from those who were not hospitalised.

## Methods

### Study design and participants

The DECIDE study was a prospective, longitudinal cohort study aiming to examine the effects of delirium upon cognition [10]. To account for baseline cognitive function, DECIDE was nested within the Cognitive Function and Ageing Study II–Newcastle cohort (CFAS II–Newcastle). CFAS II is a large, population-based cohort of people aged ≥65 years from three geographical areas in the UK: Cambridgeshire, Nottingham and Newcastle upon Tyne [11]. For wave 1 of CFAS II-Newcastle, 2,582 participants were interviewed between February 2009 and November 2011. Two years later, 1751 of these participants were re-interviewed for wave 2.

Between 5 January 2016 and 5 January 2017, surviving participants from CFAS II–Newcastle, with and without dementia, were invited to take part in DECIDE on admission to hospital as an emergency or electively. The study team were alerted to admissions by a Recurring Admission Patient Alert applied to the electronic medical records of CFAS II-Newcastle participants. If the participant themselves lacked capacity, an appropriate personal consultee was requested to provide written confirmation of willingness to participate. Once recruited, participants were seen on each subsequent hospital admission during the study period. Participants were excluded if they lacked capacity to consent and we were unable to identify or contact an appropriate personal consultee; they were receiving end-of-life care; they were being isolated for infection control reasons; or if they were expected to be in hospital for fewer than 24 h.

All participants from DECIDE were invited for follow-up 1 year after their hospital admissions to complete the CFAS II interview for a third time (wave 3). In addition, 100 control participants from CFAS II-Newcastle who had not been admitted to hospital during 2016, matched for age, sex and years of education to DECIDE participants, were also interviewed.

### Exposures

During 2016, consented individuals were reviewed daily for delirium during hospital admissions using a standardised approach based on DSM 5, described within the published DECIDE protocol [10, 12]. If consented participants could not be seen for any reason during their admissions, medical notes were reviewed using a validated tool to look for evidence of delirium [13]. Delirium status during the preceding year in those not admitted to hospital was ascertained during the home interview using the Informant Assessment of Geriatric Delirium Scale (I-AGeD) with a cut-off >4 indicating delirium [14].

### Outcomes

The primary outcome used for this work was the Mini-Mental State Examination (MMSE) score recorded as part of the CFAS II interviews at waves 1, 2 and 3. The full content of the standardised interviews is available online (http://www.cfas.ac.uk/). Delirium symptoms were assessed as part of the CFAS II interviews. Using an algorithm based upon these responses, designed specifically for use within this cohort, delirium status at the time of the wave 3 interview was determined [15].

### Statistical analysis

Participants were divided into three groups: no hospitalisation, hospitalisation without delirium and hospitalisation with delirium. Between-groups differences, when evaluating all three groups as a whole, were evaluated using a one-way analysis of variance or Kruskall–Wallis test, as appropriate. Independent t-test, Mann–Whitney or chi-squared test were used when comparing two groups. Bonferroni correction was used for multiple comparisons. Within R, *lme4* was used to perform linear mixed effects modelling (LMEM) to determine change in MMSE using piecewise linear growth from wave 1 and wave 2 of CFAS II assessments (time 1), and from wave 2 to DECIDE follow-up wave 3 (time 2). This was to account for a change point after wave 2, where participants were recruited to the DECIDE study following admissions to hospital in 2016. For comparison, change in MMSE was also modelled with time as a linear function (from waves 1, 2 and 3, Supplementary Table 2). This form of multilevel modelling is suitable for longitudinal data analysis because of its ability to handle missing data, as it does not exclude subjects with missing data from the analysis. Rate of change was modelled for all participants with baseline age, years of education, sex, time, number of hospital admissions and delirium diagnosis as fixed effects, in addition to interactions with time and age (age x time), hospital admissions (number of admissions x time) and delirium (delirium x time). A random intercept and slope model was used, which varied at the participant level and accounted for by time variation. This analysis was repeated to include duration of hospital stay (total numbers of days spent in hospital in 2016) to determine whether length of hospital stay has an impact on cognitive decline. Statistical analyses were performed using STATA Version 16 and R software (Version 3.6.0; R Foundation for Statistical Computing, Vienna, Austria).

## Results

A total of 363 CFAS II-Newcastle participants were admitted to hospital during 2016. Of these, 280 were eligible for the DECIDE study and 205 were recruited (73%) [16]. In total, 82 participants (40%) had at least one episode of delirium during the 1 year study period.

At 1 year follow-up for DECIDE (mean of 6.8 ± 0.7 years since CFAS II wave 1 interview), 38 participants had died; 135 of the remaining 167 participants completed assessments (81%) with 32 participants refusing follow-up. Three participants, who all experienced delirium, were unable to complete the MMSE due to advanced dementia. Participants who did not return for 1 year follow-up had significantly poorer MMSE scores at wave 1 (mean = 26.5 ± 2.8 vs. 27.3 ± 2.8, respectively, *P* = 0.020) and wave 2 (mean = 25.7 ± 3.2 vs. 26.7 ± 3.2, respectively, *P* = 0.015), but did not differ in terms of baseline age, sex or years of education (*P* > 0.05 for all, Supplementary Table 1). No participants met the criteria for delirium at the time of the wave 3 interview.

A total of 100 of the 187 control participants approached from CFAS II-Newcastle, with no hospital admissions during 2016, were recruited and interviewed (53%); all controls completed the MMSE. No controls experienced delirium according to the i-AGeD tool.

**Figure 1:**
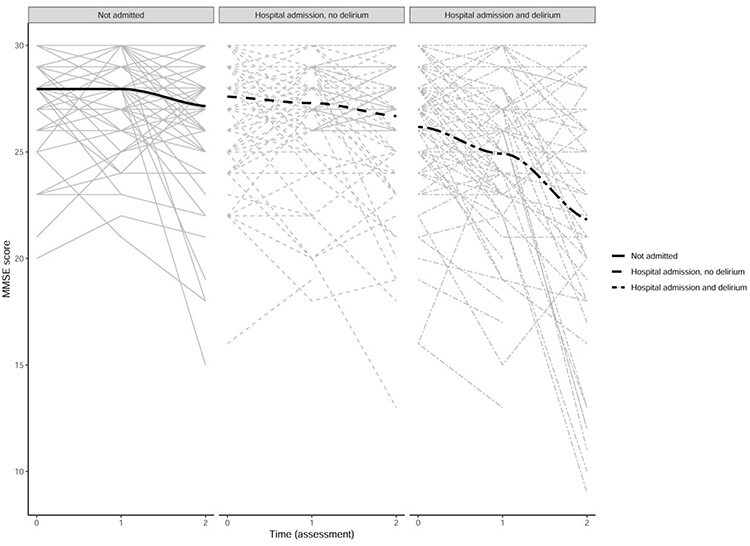
Line graphs comparing MMSE change over time in participants with no hospitalisation (not admitted), hospitalisation without delirium and hospitalisation with delirium. Time (assessment): 0 = baseline assessment (wave 1), 1 = first follow-up assessment (wave 2), 2 = second follow-up assessment (wave 3). *Delirium status ascertained during a 1 year period between wave 2 and wave 3.*

Table 1 shows the baseline characteristics of the sample stratified by group: hospitalisation and delirium, hospitalisation no delirium and no hospitalisation. Participants who developed delirium were significantly older and more cognitively impaired than those who did not (Bonferroni correction *P* < 0.017 for all, Table 1). Participants with delirium spent more days in hospital compared to those without delirium (*P* < 0.010). There was no significant difference in baseline or follow-up MMSE and a similar rate of decline in participants with no hospital admissions compared to those with hospital admissions but no delirium (*P* > 0.05 for all). By comparison, participants with a hospital admission and delirium have a lower baseline MMSE (*P* < 0.017). Trajectories of MMSE change between these three groups are illustrated in Figure 1. All groups show cognitive decline between baseline (wave 1) and follow-up (wave 3). Participants with delirium had a steeper rate of cognitive decline between waves 2 and 3 compared to those who were not admitted to hospital and those who were admitted to hospital but did not have delirium.

**Table 1:** Characteristics of participants within the three groups—hospitalisation and delirium, hospitalisation without delirium and no hospitalisation during 2016

	Hospitalisation, delirium (n = 48)	Hospitalisation, no delirium (n = 87)	No hospitalisation (n = 100)	F/Z/χ^2^	*P* value
Age at wave 1, years	79.1 (6.0)	74.5 (5.9)	75.6 (5.5)	10.4	**<0.001** a,b
Female, n (%)	26 (54.2%)	48 (55.2%)	55 (55.0%)	0.0	0.989
Years of education	10.1 (1.7)	10.5 (1.9)	10.8 (2.6)	4.3	0.111
Number of hospital admissions during 2016	2.1 (0.1)	2.1 (.02)	N/A	-1.5	0.125
Total number of days spent in hospital during 2016, median (IQR)	23.0 (21.5)	5.0 (7.0)	N/A	-6.3	**<0.001**
MMSE score wave 1	26.2 (3.3)	27.9 (2.2)	28.0 (1.9)	15.7	**<0.001**a,b
MMSE score wave 2	25.2 (3.9)	27.4 (2.5)	28.0 (1.9)	25.1	**<0.001** a,b
MMSE score at follow-up interview (wave 3)^†^	21.8 (5.8)	26.7 (3.2)	27.2 (2.9)	35.5	**<0.001** a,b

*Data presented are mean (SD) unless otherwise stated, post hoc Bonferroni correction for three group comparison at P* < *0.017, a Hospitalisation, delirium vs. Hospitalisation, no delirium, b Hospitalisation, delirium vs. No hospitalisation, No significant differences were found between Hospitalisation, no delirium vs. No hospitalisation. MMSE = Mini-Mental State Examination, IQR = interquartile range. † n = 3 participants with delirium were unable to complete MMSE at follow-up due to advanced dementia.*

LMEM determined associations with MMSE from baseline to follow-up, with a change point after wave 2, controlling for age, sex and years of education (Table 2). The number of hospital admissions was not associated with MMSE score or cognitive decline (*P* > 0.05 for all). Participants with delirium had poorer cognition at all time points (β = −1.1, *P* = 0.002). Between wave 1 and 2 (time 1), participants who developed delirium had a faster rate of cognitive decline compared to those without delirium by 0.9 points on the MMSE (β = −0.9, *P* = 0.004). This rate of decline increased to 2.2 points on the MMSE between wave 2 and wave 3 (time 2) compared to those who did not have delirium during the DECIDE study (β = −2.2, *P* < 0.001).

**Table 2:** Predictors of cognitive decline using the MMSE over time using piece-wise growth

Variables in model	β	SE	df	t-value	*P* value	
Intercept	29.4	2.0	407.4	14.6	**<0.001**	^*^^*^^*^
Age (years)	-0.05	0.02	428.4	-2.1	**0.033**	^*^
Sex (female)	0.2	0.3	304.3	0.8	0.445	
Education (years)	0.2	0.1	304.7	3.7	**<0.001**	^*^^*^^*^
Time 1	2.2	1.5	305.0	1.4	0.160	
Time 2	5.8	2.7	272.3	2.1	**0.034**	^*^
Delirium	-1.1	0.4	429.2	-3.1	**0.002**	^*^^*^
No. hospital admissions	-0.1	0.1	429.3	-0.7	0.502	
Age x Time 1	-0.03	0.02	305.0	-1.5	0.145	
Age x Time 2	-0.09	0.04	271.9	-2.4	**0.017**	^*^
Delirium x Time 1	-0.9	0.3	305.0	-2.9	**0.004**	^*^^*^
Delirium x Time 2	-2.2	0.6	265.4	-3.7	**<0.001**	^*^^*^^*^
No. hospital admissions x Time 1	-0.1	0.1	305.0	-1.2	0.244	
No. hospital admissions x Time 2	-0.1	0.2	267.4	-0.8	0.447	

Significant results highlighted in bold. ^*^P < 0.05, ^*^^*^P < 0.01, ^*^^*^^*^P < *0.001*

*MMSE = Mini-Mental State Examination. Time 1 = wave 1 to wave 2 assessments, Time 2 = wave 2 to wave 3 assessments.*

*Delirium status ascertained during a 1 year period during Time 2 (wave 2 to wave 3 assessments).*

As participants with delirium spent significantly longer in hospital compared to those who did not have delirium, this analysis was repeated to include the total number of days spent in hospital (Supplementary Table 3). The total duration of hospital stay was not significantly associated with cognitive decline at time 1 or time 2 (*P* > 0.05 for both). However, as shown in the previous analysis, participants with delirium had a faster rate of cognitive decline compared to those without delirium (time 1: β = −0.7, *P* = 0.046; time 2: β = −2.1, *P* = 0.001).

## Discussion

In a representative, population-based sample of older people, hospital admission without delirium was not associated with cognitive decline compared with a control group without hospitalisation. However, having delirium during hospital admission was associated with a 2.2 points reduction in global cognitive function assessed using the MMSE compared to those without delirium. Taken together, these results suggest that delirium may be a key component of the cognitive decline observed after hospitalisation.

Previous studies demonstrating a link between hospitalisation and cognitive decline have not considered delirium [1–6]. It is possible that the negative impact of hospitalisation on cognition demonstrated in these studies was due to delirium. Our results provide new insights into the relationship between hospitalisation and cognitive decline in older people by demonstrating that not all were at increased risk of cognitive decline and that delirium during a hospital admission seemed to differentiate between those at increased risk of cognitive decline and those who were not. This was true across all hospital settings and all types of admission, as the DECIDE study captured all elective and emergency admissions over 24 h duration, despite previous studies demonstrating worse outcomes in non-elective, critical illness and medical admissions [1–4].

As opposed to Sprung *et al*. [2] our study found that number of hospitalisations was not associated with cognitive decline . We additionally found that although the participants with delirium spent more days in hospital during the 1 year study period, total duration of hospital stay was not associated with cognitive decline in our models. This difference may be due to the inclusion of delirium as a variable within our modelling or due to the considerably longer period over which admissions were captured and followed up by Sprung *et al*.

It is possible that hospitalisation itself may have effects in people with delirium as a change of environment can precipitate or worsen delirium, and the management of delirium at home may be associated with improved outcomes. A study of community-managed delirium would be necessary to examine this but would be extremely challenging as participants would need to be assessed in the home to determine and account for differences in duration and severity of delirium in those managed at home compared with those managed in hospital.

The associations we have demonstrated between baseline cognitive impairment and delirium, along with delirium and subsequent cognitive decline, are in line with previous population-based studies in older people [7, 8]. It is not known whether delirium is specifically causing the cognitive decline described in this and other studies, or simply a manifestation of acute, severe illness and/or underlying co-morbidities. The DECIDE study was the first study to robustly attempt to address this by demonstrating that delirium was strongly associated with cognitive decline, even when accounting for illness severity, frailty and comorbidity [16]. The DECIDE study strengthened the argument for a causative relationship by showing that greater delirium exposure, in terms of more days with delirium, more episodes of delirium and more severe delirium were all associated with worse cognitive outcomes. The underlying mechanism by which delirium may cause worsening cognition is not known, but could include stress responses, effects of medication and multiplicative effects on underlying neuropathology [17].

### Study strengths and limitations

A strength of DECIDE was the prospective assessments during 2016 and the standardised approach to delirium ascertainment. Nesting DECIDE within an existing, well-characterised, population-based cohort with known baseline cognition was also an advantage, as baseline cognition could be accounted for when quantifying cognitive outcomes in a representative sample. The study had a number of limitations. First, while it is the biggest prospective study of its kind to date, it is nonetheless a single-centre sample and our sample size was smaller than previous population-based studies exploring the effects of hospitalisation [6]. The DECIDE study was powered to look at the effects of delirium rather than comparing the group hospitalised without delirium with those not hospitalised. It remains possible that a larger sample size may have detected smaller changes in cognition in those hospitalised without delirium. The sample size also limited the subgroup analysis which could be performed, including comparing cognitive outcomes in incident versus prevalent delirium. Secondly, the data only capture hospitalisations and delirium during 2016 and it is possible that a number of people in the ‘no hospitalisation’ group could have been admitted to hospital or had delirium in preceding years. However, misclassification of this kind suggests our estimates are likely to be conservative. Finally, there was a considerable gap between wave 2 interviews, completed in 2013, and follow-up interviews in 2017 (wave 3), although we included follow-up duration and by time variability in our models.

### Clinical significance

Despite being present in at least 15% of older people in hospital, delirium is often missed by clinical teams [18]. As well as being associated with considerable mortality and worse functional outcomes [19], our study demonstrates that delirium is a key marker of adverse cognitive outcomes following hospitalisation. This emphasises the importance of recognising delirium. Previous work has shown that delirium is modifiable, but further work is required focused on whether delirium intervention, in the form of both prevention and treatment, prevents cognitive decline [9]. It is also possible that rehabilitation following delirium may ameliorate some of the effects of delirium but more studies on interventions to promote recovery from delirium are needed [20].

## Conclusions

Hospital admission without delirium was not associated with cognitive decline, with cognitive trajectories equivalent to those without a hospital admission. Delirium during hospitalisation was associated with significant cognitive decline, suggesting that it is delirium during hospitalisation rather than hospitalisation alone that is a risk factor for future cognitive decline. This emphasises the urgent need for dementia prevention studies that focus on delirium intervention.

## Supplementary Material

aa-20-1341-File002_afab068
