# Supplementary material for: Hospitalisation without delirium is not associated with cognitive decline in a population-based sample of older people—results from a nested, longitudinal cohort study
Source: Age Ageing. 2021 May 3;50(5):1675–81. doi: 10.1093/ageing/afab068 (PMC8437075; doi:10.1093/ageing/afab068)
Supplement: aa-20-1341-File002_afab068 [file aa-20-1341-file002_afab068.docx]

Hospitalisation without delirium is not associated with cognitive decline in a population-based sample of older people – results from a nested, longitudinal cohort study

**SUPPLEMENTARY DATA**

**Supplementary Table 1: Characteristics of participants who did and did not return for follow up assessments (wave 3)**

|  | **Completers (n=135)** | **Non--completers (n=70)** | **F/Z/χ^2^** | **p-value** |
| --- | --- | --- | --- | --- |
| Age at wave 1, years | 76.1(6.4) | 77.3(6.8) | -1.2 | 0.235 |
| Female, n(%) | 74(54.8) | 35(50.0) | 0.4 | 0.512 |
| Years of education | 10.4(1.8) | 10.6(2.9) | -0.4 | 0.706 |
| MMSE score wave 1 | 27.3(2.8) | 26.5(2.8) | -2.3 | **0.020** |
| MMSE score wave 2 | 26.7(3.2) | 25.7(3.2) | -2.4 | **0.015** |

Data presented are mean (SD) unless otherwise stated. Non-completers: n=38 died, n=32 did not return for follow-up assessment.

MMSE = Mini-Mental State Examination.

**Supplementary Table 2:** **Predictors of cognitive decline using the MMSE over time**

| **Variables in model** | **β** | **SE** | **df** | **t-value** | **p-value** |  |
| --- | --- | --- | --- | --- | --- | --- |
| Intercept | 28.8 | 1.9 | 311.6 | 14.9 | **<0.001** | *** |
| Time | 3.8 | 1.3 | 290.7 | 3.0 | **0.003** | ** |
| Age (years) | -0.04 | 0.02 | 301.4 | -1.9 | 0.063 |  |
| Sex (female) | 0.2 | 0.3 | 304.3 | 0.7 | 0.498 |  |
| Education (years) | 0.2 | 0.1 | 304.7 | 3.8 | **<0.001** | *** |
| Delirium | -1.0 | 0.3 | 304.3 | -2.8 | **0.006** | ** |
| No. hospital admissions | -0.1 | 0.1 | 303.3 | -0.7 | 0.481 |  |
| Age x Time | -0.06 | 0.02 | 291.9 | -3.3 | **0.001** | ** |
| Delirium x Time | -1.4 | 0.3 | 320.9 | -5.1 | **<0.001** | *** |
| No. hospital admissions x Time | -0.1 | 0.1 | 311.5 | -1.4 | 0.171 |  |

Significant results highlighted in bold. MMSE = Mini-Mental State Examination. *p<0.05, **p<0.01, ***p<0.001

**Supplementary Table 3: Predictors of cognitive decline using the MMSE over time using piece-wise growth**

| **Variables in model** | **β** | **SE** | **df** | **t-value** | **p-value** |  |
| --- | --- | --- | --- | --- | --- | --- |
| Intercept | 29.3 | 2.0 | 406.5 | 14.5 | **<0.001** | *** |
| Age (years) | 2.2 | 1.5 | 305.0 | 1.4 | 0.151 |  |
| Sex (female) | 5.6 | 2.7 | 272.1 | 2.0 | **0.043** | * |
| Education (years) | -0.1 | 0.0 | 427.4 | -2.1 | **0.036** | * |
| Time 1 | 0.2 | 0.3 | 304.4 | 0.8 | 0.401 |  |
| Time 2 | 0.2 | 0.1 | 304.8 | 3.7 | **<0.001** | *** |
| Delirium | -1.2 | 0.4 | 428.7 | -2.9 | **0.003** | ** |
| No. hospital admissions | 0.0 | 0.0 | 429.0 | -0.1 | 0.917 |  |
| Age x Time 1 | 0.0 | 0.0 | 305.0 | -1.5 | 0.131 |  |
| Age x Time 2 | -0.1 | 0.0 | 271.8 | -2.3 | **0.021** | * |
| Delirium x Time 1 | -0.7 | 0.3 | 305.0 | -2.0 | **0.046** | * |
| Delirium x Time 2 | -2.1 | 0.6 | 267.0 | -3.3 | **0.001** | ** |
| No. days in hospital x Time 1 | 0.0 | 0.0 | 305.0 | -1.8 | 0.071 |  |
| No. days in hospital x Time 2 | 0.0 | 0.0 | 260.5 | -0.9 | 0.391 |  |

Significant results highlighted in bold. *p<0.05, **p<0.01, ***p<0.001

MMSE = Mini-Mental State Examination. Time 1 = wave 1 to wave 2 assessments, Time 2 = wave 2 to wave 3 assessments
